# Tocotrienol rich fraction supplementation improved lipid profile and oxidative status in healthy older adults: A randomized controlled study

**DOI:** 10.1186/1743-7075-8-42

**Published:** 2011-06-24

**Authors:** Siok-Fong Chin, Johari Ibahim, Suzana Makpol, Noor Aini Abdul Hamid, Azian Abdul Latiff, Zaiton Zakaria, Musalmah Mazlan, Yasmin Anum Mohd Yusof, Aminuddin Abdul Karim, Wan Zurinah Wan Ngah

**Affiliations:** 1 Department of Biochemistry, Faculty of Medicine, Universiti Kebangsaan Malaysia, Kuala Lumpur, Malaysia; 2 Department of Anatomy, Faculty of Medicine, Universiti Kebangsaan Malaysia, Kuala Lumpur, Malaysia; 3 Department of Physiology, Faculty of Medicine, Universiti Kebangsaan Malaysia, Kuala Lumpur, Malaysia

## Abstract

**Background:**

Vitamin E supplements containing tocotrienols are now being recommended for optimum health but its effects are scarcely known. The objective was to determine the effects of Tocotrienol Rich Fraction (TRF) supplementation on lipid profile and oxidative status in healthy older individuals at a dose of 160 mg/day for 6 months.

**Methods:**

Sixty-two subjects were recruited from two age groups: 35-49 years (n = 31) and above 50 years (n = 31), and randomly assigned to receive either TRF or placebo capsules for six months. Blood samples were obtained at 0, 3^rd ^and 6^th ^months.

**Results:**

HDL-cholesterol in the TRF-supplemented group was elevated after 6 months (p < 0.01). Protein carbonyl contents were markedly decreased (p < 0.001), whereas AGE levels were lowered in the > 50 year-old group (p < 0.05). Plasma levels of total vitamin E particularly tocopherols were significantly increased in the TRF-supplemented group after 3 months (p < 0.01). Plasma total tocotrienols were only increased in the > 50 year-old group after receiving 6 months of TRF supplementation. Changes in enzyme activities were only observed in the > 50 year-old group. SOD activity was decreased after 3 (p < 0.05) and 6 (p < 0.05) months of TRF supplementation whereas CAT activity was decreased after 3 (p < 0.01) and 6 (p < 0.05) months in the placebo group. GPx activity was increased at 6 months for both treatment and placebo groups (p < 0.05).

**Conclusion:**

The observed improvement of plasma cholesterol, AGE and antioxidant vitamin levels as well as the reduced protein damage may indicate a restoration of redox balance after TRF supplementation, particularly in individuals over 50 years of age.

## Background

Dietary and supplemental sources of vitamin E isoforms have been demonstrated to possess unique properties that can influence critical pathways involved in cancer [1,2], cardiovascular [3,4] and neurodegenerative disease [5,6] development. Recent studies have identified tocotrienol, the lesser known isomer of vitamin E as the more effective compound in providing such protection in comparison to the well-established tocopherol [7-14]. The most promising function of tocotrienol has been reported in neuroprotection [14,15] and stroke prevention with the latter being attributed to the lipid-corrective properties of tocotrienol [11,13]. However, most data were generated from *in vitro *studies or by using animal models. To date, very limited data available from human intervention study particularly involving supplemental vitamin E and its fundamental effects on diseases and ageing.

Recent interest has focussed on finding compounds that could intervened the chemical processes underlying age-related degenerative diseases in which has been demonstrated to be also responsible for the ageing phenomena [16,17]. Although vitamin E supplementation has yielded equivocal results in human intervention studies largely on a short term basis [18-20], supplementation studies measuring benefits to cardiovascular end points are still controversial with some showing no benefits and some even detrimental effects [21-23]. Studies are warranted to elucidate the underlying mechanism on the effects of vitamin E in preventing or treating these diseases that may explain the observed effects.

Most vitamin E supplements available in the market usually contain only alpha tocopherol. However, recently with the availability of tocotrienols commercially, the uses of tocotrienol-containing supplement are more widespread. There has been a paradigm shift to vitamin E supplementation where all isomers of vitamin E are recommended rather than alpha tocopherol alone [24]. However, it has yet to be determined whether supplementation with a mixture of vitamin E isomers containing high tocotrienol fractions will affect biochemical parameters and other blood indices and the possible underlying mechanism. We are interested in investigating age-associated biochemical changes with tocotrienol enriched vitamin E supplementation and identifying the specific oxidation pathways involved.

Different aged individuals respond differently to various food and vitamin supplementation [25]. Needs for supplement may be different with the different age where older people may benefit from supplementation as they were reported to have lower antioxidant levels [26].

We previously reported that TRF supplementation decreased DNA damage in healthy older adults, mostly in those over 50 years of age, and that the levels of damage were associated with age [27]. In the present work, changes in lipoprotein-lipid profile, protein carbonyl content, advanced glycosylation end products (AGEs), malondialdehyde (MDA) levels, levels of the antioxidant vitamins E and C, and antioxidant activities of superoxide dismutase (SOD), catalase (CAT) and glutathione peroxidase (GPx) were measured in a randomized, double-blinded, placebo-controlled intervention study of TRF supplementation. We also studied the association between oxidative biomarkers and antioxidant levels to further elucidate the oxidant-antioxidant balance with increasing age.

## Methods

### Study design

Healthy volunteers were recruited through screening of a population study on oxidative stress and ageing. Selected individuals were aged 35 years and older, non-smokers, not pregnant, not taking any vitamin supplements or minerals, alcohol, or drugs, and free of cardiac, hepatic, renal or any other chronic diseases. Adult females were recruited as the compliance from this gender is higher and mostly are non-smokers. Results of a pre-study consisting a full physical examination, previous medical history, blood chemistry and haematology were used to confirm suitability. Sixty-two subjects were recruited from two age groups, 35-49 years (n = 31) and over 50 years (n = 31), and randomly assigned to receive either Tocotrienol Rich Fraction (TRF) capsules (160 mg/day) daily in a single evening dose or an identical placebo for a period of six months. All subjects were requested to consume the capsules after dinner to ensure proper absorption [28,29] and encouraged to maintain their usual lifestyle throughout the study period. The commercially prepared TRF (Tri E^® ^Tocotrienol) is a palm-based vitamin E consisted of approximately 74% tocotrienols and 26% tocopherol in soft gelatine capsules containing palm superolein oil, and was supplied by Sime Darby Bioganic Sdn. Bhd. (previously known as Golden Hope Bioganic, Selangor, Malaysia). Each capsules contained approximately 70.4 mg α-tocotrienol, 4.8 mg β-tocotrienol, 57.6 mg γ-tocotrienol, 33.6 mg δ-tocotrienol and 48 mg α-tocopherol. The capsules given throughout the study were from the same lot and provided loosely in plastic containers. The placebo capsules contained only palm superolein oil. In addition to plasma samples for vitamin E levels, compliance was assessed by capsule counts at each 3-month interval. The amount of allotted and returned capsules for each participant is recorded during the interval visits. The treatment was double blinded throughout the study period until all data were collected, after which the randomisation code was broken. The protocol of the study was approved by the Research and Ethics Committee of the Faculty of Medicine, Universiti Kebangsaan Malaysia. Written informed consent was obtained. Subject demographics are summarised in Table 1.

**Table 1:** Baseline and intervention characteristics of the study groups

	Placebo (n = 30)		TRF (n = 32)	
	
	35-49 years	> 50 years	35-49 years	> 50 years
Age (years)	44.7 ± 0.9 ^a^	59.1 ± 1.7 ^a^	44.5 ± 1.0 ^b^	56.1 ± 1.2 ^b^
Blood pressure (mmHg)				
Systolic				
Baseline (0 month)	121 ± 2.8 ^c^	137 ± 3.6 ^c^	121 ± 3.5 ^d^	131 ± 3.3 ^d^
3 months	123 ± 2.4 ^f^	137 ± 5.1 ^f^	121 ± 3.4	131 ± 3.5
6 months	120 ± 2.5 ^g^	135 ± 5.0 ^g^	122 ± 3.6	132 ± 3.3
Diastolic				
Baseline (0 month)	75.4 ± 1.9 ^e^	82.0 ± 2.7 ^e^	80.6 ± 2.8	81.3 ± 2.6
3 months	73.7 ± 1.7	79.2 ± 2.9	79.2 ± 2.6	82.3 ± 2.6
6 months	75.7 ± 2.8	79.2 ± 3.3	79.0 ± 3.1	82.8 ± 1.9
Pulse (beat/minute)				
Baseline (0 month)	73 ± 2	73 ± 3	70 ± 3	70 ± 2
3 months	70 ± 2	72 ± 2	71 ± 3	71 ± 2
6 months	71 ± 2	71 ± 3	69 ± 3	72 ± 2
Body mass index (kg/m^2^)				
Baseline (0 month)	27.5 ± 1.2	26.3 ± 0.6	26.3 ± 0.7	27.7 ± 1.3
3 months	27.5 ± 1.2	25.0 ± 1.0	25.6 ± 0.6	27.4 ± 1.2
6 months	26.8 ± 1.2	25.2 ± 1.0	25.7 ± 0.7	27.1 ± 1.1
Fasting blood glucose (mmol/L)				
Baseline (0 month)	5.53 ± 0.33	5.34 ± 0.13	5.74 ± 0.30	5.49 ± 0.10
3 months	5.40 ± 0.44	5.37 ± 0.10	5.59 ± 0.22	5.24 ± 0.15
6 months	5.55 ± 0.49	5.28 ± 0.14	5.42 ± 0.19	5.47 ± 0.11

Values are expressed as mean ± SEM.

Same letters indicate significant difference between 35-49 and > 50 year-old groups.

a: p < 0.001, b: p < 0.001, c: p < 0.001, d: p < 0.05, e: p < 0.05, f: p < 0.001, g: p < 0.01

### Sample collection

Blood sampling was performed at baseline (month 0), 3 months and 6 months of supplementation. Venous blood samples were drawn from fasting subjects into lithium heparin-coated and K_2_EDTA-containing tubes (BD Vacutainer, Becton, Dickinson and Company, Franklin Lakes, NJ, USA) for plasma extraction while into a plain tube for serum extraction. Plasma and serum were immediately separated by centrifugation at 3000 g for 10 minutes. The obtained packed erythrocytes were washed three times with 0.9% sodium chloride solution. Heparinized plasma aliquots were separated for lipid profiling as well as vitamin C and vitamin E determination. Plasma-EDTA aliquots were used for protein carbonyl and MDA quantification whereas serum aliquots were used for AGE product testing. Washed erythrocytes were used for determination of antioxidant activities of SOD, CAT and GPx. Plasma samples for vitamin C determination were deproteinised with 5% perchloric acid and centrifuged at 1000 g for 2 minutes, and the resulting clear supernatant was transferred into new tubes. Lipid profile testing was done immediately whereas other samples were frozen at -80°C until further analysis.

### Lipid profile determination

Plasma cholesterol and triglyceride concentrations were determined using enzymatic colorimetric kits (Roche diagnostics GmbH, Indianapolis, USA) on a Hitachi 747 Automatic Analyzer (Boehringer Mannheim, Germany). High density lipoprotein-cholesterol (HDL-C) was measured using the precipitation method whereas low density lipoprotein-cholesterol (LDL-C) was calculated using Friedewald's formula.

### Plasma vitamin E determination

Plasma tocopherol and tocotrienol were determined by HPLC. Briefly, stored plasma samples were thawed and aliquots of 200 µl plasma and 50 µl 95% ethanol containing 10 µg/ml butylated hydroxytoluene (Sigma Chemical Co., St. Louis, MO, USA) were pipetted into tubes, covered and mixed vigorously for 5 seconds. 1 ml of absolute ethanol (Merck KGaA, Darmstadt, Germany) was then added to each tube. The tubes were covered and mixed again before being centrifuged at 1500 g for 15 minutes at 18°C. The bottom pellet was carefully removed with a spatula. Hexane, 3 ml (Merck KGaA, Darmstadt, Germany) were then added to each tube and mixed vigorously for 5 minutes. The samples were then centrifuged at 1500 g for 15 minutes at 18°C. After centrifugation, a portion of the upper layer (2.5 ml) was carefully removed, placed in new tubes and vacuum-evaporated for 40 minutes. The dried sample residue was then reconstituted in 100 µl HPLC grade hexane (Merck KGaA, Darmstadt, Germany), vortexed, and passed through a 0.45 µm filter to remove any non-dissolved particles. The samples were then transferred to amber vial inserts with care taken to avoid air bubbles and analysed by HPLC using a Shimadzu RF-10A XL fluorescence detector (Shimadzu Corporation, Kyoto, Japan) at an excitation wavelength of 294 nm and an emission wavelength of 330 nm. Isomers α-tocopherol, γ-tocopherol, α-tocotrienol, γ-tocotrienol and δ-tocotrienol were separated on a 250 mm × 4.6 mm, 5 µm Allsphere Silica column (Alltech Associated, Inc, IL, USA) and eluted with a mobile phase of 99:1 (v/v) hexane-isopropanol at a flow rate of 1.5 ml per minute. The identity of each compound was confirmed by co-elution with spiked standard. All external standards used were obtained from Malaysian Palm Oil Board (MPOB), Malaysia. The peaks were quantified and integrated with Shimadzu Class-VP™ version 6.1 LC Workstation software (Shimadzu Corporation, Kyoto, Japan).

### Plasma vitamin C determination

The measurement of plasma vitamin C (ascorbic acid) was performed using HPLC as described by Pachla and Kissinger [30]. Plasma ascorbic acid was chromatographically separated on a 25 cm VYDAC^® ^Genesis C-18 4 µm column (Grace Davison, USA) using a mobile phase containing 0.04 M sodium acetate, 0.48 mM disodium EDTA, 2.9 mM tetrabutylammonium hydroxide and 1.4% methanol at pH 4.75. Detection was performed on an electrochemical detector (Gilson model 142, Gilson Medical Electronics S.A., Villiers-le-Bel, France) set at an applied potential of +700 mV and referenced to an Ag/AgCl electrode, with a flow rate of 1 ml/min and sensitivity of 100 nA/V on a Gilson HPLC set (Gilson Medical Electronics S.A., Villiers-le-Bel, France). Assay calibration was performed for each run using six concentrations of the calibrator (0, 2, 4, 6, 8, 10 and 12 mg/L). Stored deproteinised plasma samples were thawed, added with 5 mg/L 3,4-dihydroxybenzylamine (Sigma Chemical Co., USA) and spiked with 5 mg/L ascorbic acid (Sigma Chemical Co., St. Louis, MO, USA). 3,4-dihydroxybenzylamine was added to all samples and calibrators as an internal standard whereas uric acid Raichem™ (Reagents Applications Inc., USA) was injected separately for peak identification. All samples were filtered through a 0.45 µm membrane before being subjected to HPLC and all peaks were baseline separated. Ascorbic acid, 3,4-dihydroxybenzylamine and uric acid peaks were resolved and detected within 11 minutes of run time and were identified using the retention times of calibrators. Peaks were integrated using peak-area ratios of ascorbic acid to 3,4-dihydroxybenzylamine.

### Plasma protein carbonyl determination

Carbonyl content in oxidatively-modified proteins was assayed using the Cayman Chemical protein carbonyl assay kit (Cayman Chemical Company, Ann Arbor, MI, USA) based on the method of Levine *et al. *[31] by following the manufacturer instructions. Briefly, 2,4-dinitrophenyl-hydrazine (DNPH) reacts with protein carbonyls, forming a Schiff base to produce the corresponding hydrazone. The protein-hydrazone produced was quantified at an absorbance of 370 nm with an extinction coefficient of 22,000 M^-1^cm^-1^. Each sample was assayed with a parallel control and the concentration of carbonyls was determined after correction with the respective control. The carbonyl content was then standardized against the protein concentration in the sample and expressed as nmol carbonyl per mg protein. The amount of protein was calculated from a bovine serum albumin (Sigma Chemical Co., St. Louis, USA) standard curve (0.25-2.0 mg/ml) read at 280 nm.

### Serum AGE determination

Serum advanced glycosylation end products (AGEs) was measured with an in-house competitive enzyme immunoassay technique developed by Wan Nazaimoon and Khalid [32]. Briefly, microtitre wells were coated with AGE-BSA at 8 µg/ml followed by an overnight incubation with 20 µl of the prediluted sample (1:6) and 80 µl anti-AGE-KLH (1:8000). HRP-labelled goat anti-rabbit (1:3000) was used as the secondary antibody and 3,5',5,5'-tetramethylbenzidine dihydrochloride (Sigma Chemical Co., St. Louis, USA) as the substrate. The colour reaction was stopped with 1.25 M sulphuric acid and the absorbance was read at 450 nm with reference at 620 nm. All samples were assayed in triplicate and assay performance was monitored using a set of in-house quality control sera containing three different levels of AGE.

### Plasma MDA determination

Plasma malondialdehyde (MDA) was determined by HPLC based on the derivatisation of MDA with 2,4-dinitrophenylhydrazine (DNPH) (Sigma-Aldrich, St. Louis, MO, USA) as described by Pilz *et al. *[33] with some modifications. Briefly, stored plasma samples were thawed and aliquots of 250 µl plasma were mixed with 50 µl 6 M sodium hydroxide (Merck, Germany). The sample mixture was incubated at 60°C for 30 minutes in a water bath. After cooling to room temperature, the hydrolysed sample was then acidified with 125 µl 35% (v/v) perchloric acid to precipitate proteins and centrifuged at 6000 g for 10 minutes at 18°C. The supernatant was transferred into fresh tubes and the sample was then derivatised with 50 µl 5 mM DNPH for 30 minutes at room temperature. Derivatised samples were used for HPLC analysis and protected from light from this step onwards.

Analytical HPLC separations were performed on a Shimadzu Chromatographic system (Shimadzu Class-VP™ version 6.1 LC Workstation software, Shimadzu Corporation, Kyoto, Japan) with a diode array detector equipped with an auto injector and operated at 310 nm on a 150 mm × 3.9 mm, 5 µm alphaBond C18 column (Alltech Associated, Inc. IL, USA). Samples and standards were eluted with a mobile phase consisting of 380 ml acetonitrile with 620 ml of distilled water, acidified with 0.2% (v/v) acetic acid and degassed at a flow rate of 0.6 ml/min. The plasma MDA level was calculated from a calibration curve prepared by acidic hydrolysis of 1,1,3,3-Tetraethoxypropane (TEP) (Sigma-Aldrich, St. Louis, MO, USA).

### Determination of erythrocyte antioxidant enzymes

SOD, CAT and GPx activities in the hemolysates were expressed as U/mg Hb. Erythrocyte Cu, Zn-SOD activity was assayed using the spectrophotometric indirect inhibition technique of Beyer and Fridovich, which is based on the ability of SOD to inhibit the photoreduction of nitro blue tetrazolium [34]. CAT activity was measured in erythrocytes using the method of Aebi [35] with hydrogen peroxide as the substrate. This method is based on the decomposition of hydrogen peroxide and measured by decreased absorbance at 240 nm. GPx activity was measured in erythrocytes using the coupled method of Paglia and Valentine with *t*-butyl hydroperoxide as the substrate [36]. Hemoglobin was assayed using the cyanmethemoglobin procedure (Eagle Diagnostics, Desoto, Texas, USA) based on the determination of cyanmethemoglobin at 540 nm.

### Statistical Analysis

Statistical analysis was performed using Statistical Package for Social Sciences (SPSS) Version 11.5 (Chicago, Illinois, USA). Mixed model analysis of variance (ANOVA) was used to compare changes from baseline to 3 and 6 months for all variables to ascertain the effects of treatment. One way analysis of variance was used to identify significant differences between age groups. The associations of all parameters were assessed by partial correlations and analyses of covariance to account for the increased proportion of women in the study group. Pearson's correlation coefficient was determined for baseline variables to determine their relationships. Mauchly's test of sphericity was used to assess the homogeneity of variance and Dunnett's test was performed to compare the means of the treatment group to the means of the control group. Bonferroni's adjustment was applied to control the inflation of Type 1 error across multiple tests. All data are presented as mean + standard error of the mean (SEM). The null hypothesis was tested using a 2-tailed α < 0.05 criterion. PS Program version 2.1.31 was used to determine the power of study in which the probability of rejecting the null hypothesis with Type I error was set at α = 0.05, given the specified sample size n = 30, a standard deviation σ = 0.104 and when the true difference in population means was δ = 0.097. The statistical power obtained from our approach was 0.9977.

## Results

### Subject characteristics

The baseline characteristics of subject age, gender, blood pressure, pulse, body mass index and fasting blood glucose in the TRF groups were similar to those in the placebo groups (Table 1). Systolic and diastolic blood pressure in the younger group (35-49 years old) were significantly lower than those in the older group (over 50 years old) while no significant differences was observed between groups regarding to pulse, body mass index (BMI) or fasting blood sugar (FBS). None of these clinical parameters showed statistical difference upon supplementation except for BMI (Table 1) where the younger group who received TRF demonstrated reductions in BMI at 3 months (p = 0.024) and 6 months (p < 0.001).

### Lipid profile

Plasma total cholesterol was within the normal range in all subjects before supplementation. There was no significant difference in lipids level between the younger and older groups at baseline (Table 2). A statistically significant effect for duration of treatment was observed in HDL-C in TRF group (F = 4.196, p = 0.020, effect size = 0.139, power = 0.713). The HDL-C level in the younger group increased significantly after 6 months of Tocotrienol supplementation (p = 0.014) as compared to the baseline, while in the > 50 year-old group, a non-significant elevation of 8% was observed. However, the plasma ratio of HDL-C to total cholesterol improved in both younger (p = 0.007) and older groups (p = 0.029) with supplementation. Values obtained for all lipid variables were within the normal range.

**Table 2:** Lipid profiles of study groups

	Age group			
	35-49 years		> 50 years	
	
	Placebo	TRF	Placebo	TRF
TC (mmol/L)				
Baseline (0 month)	5.12 ± 0.19	5.34 ± 0.18	5.30 ± 0.20	5.43 ± 0.19
3 months	4.91 ± 0.17	5.39 ± 0.16	5.39 ± 0.18	5.43 ± 0.20
6 months	5.04 ± 0.17	5.47 ± 0.15	5.34 ± 0.21	5.44 ± 0.20
HDL-C (mmol/L)				
Baseline (0 month)	1.37 ± 0.07	1.24 ± 0.07	1.35 ± 0.09	1.36 ± 0.10
3 months	1.42 ± 0.10	1.32 ± 0.06	1.47 ± 0.07	1.32 ± 0.10
6 months	1.41 ± 0.10	1.42 ± 0.07*	1.28 ± 0.07 ^a^	1.47 ± 0.07 ^a^
LDL-C (mmol/L)				
Baseline (0 month)	3.28 ± 0.18	3.40 ± 0.20	3.28 ± 0.20	3.42 ± 0.15
3 months	2.99 ± 0.17	3.45 ± 0.15	3.18 ± 0.17	3.45 ± 0.18
6 months	3.15 ± 0.17	3.40 ± 0.15	3.53 ± 0.16	3.23 ± 0.18
Triglycerides (mmol/L)				
Baseline (0 month)	1.12 ± 0.15	1.39 ± 0.20	1.41 ± 0.19	1.46 ± 0.26
3 months	1.23 ± 0.12	1.25 ± 0.17	1.58 ± 0.23	1.44 ± 0.25
6 months	1.02 ± 0.10 ^b^	1.48 ± 0.25	1.45 ± 0.25 ^b^	1.57 ± 0.30
HDL-C/TC				
Baseline (0 month)	26.58 ± 1.44	23.14 ± 1.63	26.30 ± 2.26	24.73 ± 1.56
3 months	28.53 ± 1.97	24.82 ± 1.36	27.30 ± 2.21	24.53 ± 1.81
6 months	28.89 ± 1.67	25.91 ± 1.53**	24.92 ± 1.82 ^c^	28.13 ± 1.52* ^c^

Values are expressed as mean ± SEM

Abbreviations: TC, total cholesterol; HDL-C, high-density lipoprotein-cholesterol; LDL-C, low density lipoprotein-cholesterol

Symbols indicate significant difference from baseline (0 month)

*: p < 0.05, **: p < 0.01

Same characters indicate significant difference as compared to placebo group.

a: p = 0.022, b: p = 0.022, c: p = 0.04

### Antioxidant vitamins

Plasma vitamin E levels were corrected for cholesterol levels. Increased plasma total vitamin E concentrations were evident after 3 months in the treatment group (F = 9.491, p = 0.006, effect size = 0.311, power = 0.836) but not in placebo group (Table 3). Further grouping of subjects by age (Table 4) revealed significant increases in lipid-corrected total vitamin E in both 35-49 year-old and > 50 year-old groups after 6 months (F = 10.233, p = 0.011, effect size = 0.532, power = 0.812 and F = 43.707, p < 0.001, effect size = 0.785, power = 1 respectively), but in the > 50 year-old group, levels had significantly increased as early as 3 months after starting treatment (F = 6.933, p = 0.022, effect size = 0.366, power = 0.677).

**Table 3:** Plasma antioxidant vitamins in placebo and TRF groups

	Duration					
Antioxidant vitamins	0 month		3 months		6 months	
	
	Placebo	TRF	Placebo	TRF	Placebo	TRF
Lipid-corrected total vitamin E (g/mol)	9.76 ± 0.38	9.35 ± 0.39	10.02 ± 0.20	10.44 ± 0.35**	9.26 ± 0.45	11.74 ± 0.57**
Lipid-corrected total tocopherol (g/mol)	9.16 ± 0.37	8.78 ± 0.40	9.53 ± 0.22	9.83 ± 0.37**	8.55 ± 0.41	10.96 ± 0.54**
Lipid-corrected total tocotrienol (g/mol)	0.63 ± 0.07	0.57 ± 0.06	0.54 ± 0.06	0.62 ± 0.06	0.69 ± 0.08	0.78 ± 0.07**
Vitamin C (mg/L)	5.09 ± 0.30	4.23 ± 0.26	5.86 ± 0.30*	5.29 ± 0.29**	6.07 ± 0.31**	5.14 ± 0.37**

Values are expressed as mean ± SEM.

Symbols indicate significant difference from baseline (0 month).

*: p < 0.05, **: p < 0.01

**Table 4:** Plasma antioxidant vitamins according to age group

	Age group			
Antioxidant vitamins	35-49 years		> 50 years	
	
	Placebo	TRF	Placebo	TRF
Lipid-corrected total vitamin E (g/mol)				
Baseline (0 month)	8.71 ± 0.37	8.57 ± 0.43	10.41 ± 0.50	9.89 ± 0.56
3 months	9.49 ± 0.24	9.59 ± 0.46	10.35 ± 0.25	11.03 ± 0.45*
6 months	9.04 ± 0.67	10.39 ± 0.53**	9.52 ± 0.61	12.89 ± 0.85**
Lipid-corrected total tocopherol (g/mol)				
Baseline (0 month)	8.04 ± 0.33	7.89 ± 0.45	9.84 ± 0.47	9.39 ± 0.57
3 months	8.92 ± 0.35	8.86 ± 0.46	9.92 ± 0.29	10.49 ± 0.48*
6 months	8.20 ± 0.62	9.66 ± 0.52**	8.97 ± 0.52	12.06 ± 0.79**
Lipid-corrected total tocotrienol (g/mol)				
Baseline (0 month)	0.69 ± 0.09	0.66 ± 0.08	0.58 ± 0.11	0.50 ± 0.07
3 months	0.61 ± 0.07	0.73 ± 0.11	0.46 ± 0.09	0.54 ± 0.07
6 months	0.80 ± 0.09	0.73 ± 0.09	0.55 ± 0.14	0.82 ± 0.11**
Vitamin C (mg/L)				
Baseline (0 month)	4.61 ± 0.35	3.95 ± 0.19	5.57 ± 0.47	4.50 ± 0.48
3 months	5.26 ± 0.42	5.16 ± 0.43*	6.48 ± 0.36	5.42 ± 0.39*
6 months	5.66 ± 0.32**	4.98 ± 0.63	6.66 ± 0.56	5.26 ± 0.46**

Values are expressed as mean ± SEM.

Symbols indicate significant difference from baseline (0 month).

*: p < 0.05, **: p < 0.01

Similar results were observed for plasma tocopherol concentration. Changes in plasma level of tocopherol were statistically significant with duration of treatment (F = 12.896, p < 0.001, effect size = 0.380, power = 0.995). Supplementation with TRF markedly increased lipid-corrected total tocopherol levels after 3 and 6 months in the > 50 year-old group (F = 8.043, p = 0.015, effect size = 0.401, power = 0.740 and F = 47.069, p < 0.000, effect size = 0.797, power = 1 respectively) as compared to the younger age group. The 35-49 year-old group only showed increased levels of lipid-corrected tocopherols after 6 months of supplementation (F = 9.445, p = 0.013, effect size = 0.512, power = 0.781).

Overall, lipid-corrected tocotrienol levels were significantly increased in TRF supplemented subjects when compared to baseline and placebo-receiving subjects after 6 months (F = 10.068, p = 0.005, effect size = 0.324, power = 0.857). However, further increases in tocotrienol levels were only observed in the > 50 year-old group after 6 months of treatment (F = 11.197, p = 0.006, effect size = 0.483, power = 0.866). Regardless of the treatment type, levels of lipid-corrected total tocotrienols in the older group were lower than in the younger group. With treatment, however, levels of lipid-corrected total tocotrienols in older subjects approached levels observed in younger subjects.

Plasma vitamin C concentrations increased in both groups regardless of the treatment received. Further analysis of age groups showed similar increases for all groups to varying degrees of significance.

### Antioxidant enzymes

A significant effect for duration was observed in SOD activity with TRF treatment (F = 6.838, p = 0.006, effect size = 0.229, power = 0.826) as well as in CAT and GPx activity with placebo (F = 4.185, p = 0.002, effect size = 0.160, power = 0.707 and F = 5.271, p = 0.009, effect size = 0.193, power = 0.809 respectively). SOD activity decreased after 3 and 6 months of TRF treatment (Table 5); these effects were seen in both younger and older groups, although only the > 50 year-old group reached statistical significance (Table 6). CAT activity was decreased in the placebo group after 3 months; this change was also only observed in the > 50 year-old group. Similar effects were observed for GPx, where only subjects in the > 50 year-old group showed increased activity after 6 months regardless of treatment.

**Table 5:** Erythrocyte antioxidant enzymes activity in placebo and TRF groups

	Duration					
Antioxidant enzymes	0 month		3 months		6 months	
	
	Placebo	TRF	Placebo	TRF	Placebo	TRF
Superoxide dismutase (U/mg Hb)	1.90 ± 0.09	2.29 ± 0.14	1.90 ± 0.16	1.83 ± 0.09**	1.91 ± 0.13	1.77 ± 0.09**
Catalase (U/mg Hb)	0.30 ± 0.01	0.28 ± 0.01	0.24 ± 0.01**	0.27 ± 0.01	0.27 ± 0.01	0.27 ± 0.01
Glutathione peroxidase (U/mg Hb)	0.82 ± 0.05	0.87 ± 0.04	0.87 ± 0.05	1.04 ± 0.08	1.04 ± 0.05**	1.09 ± 0.06*

Values are expressed as mean ± SEM.

Symbols indicate significant difference from baseline (0 month).

*: p < 0.05, **: p < 0.01

**Table 6:** Erythrocyte antioxidant enzymes activity according to age group

	Age group			
Antioxidant enzymes	35-49 years		> 50 years	
	
	Placebo	TRF	Placebo	TRF
Superoxide dismutase (U/mg Hb)				
Baseline (0 month)	1.85 ± 0.14	2.23 ± 0.22	1.89 ± 0.12	2.35 ± 0.20
3 months	1.95 ± 0.16	1.81 ± 0.15	1.85 ± 0.28	1.85 ± 0.12*
6 months	1.98 ± 0.24	1.74 ± 0.14	1.85 ± 0.10	1.79 ± 0.12*
Catalase (U/mg Hb)				
Baseline (0 month)	0.29 ± 0.02	0.28 ± 0.02	0.32 ± 0.02	0.28 ± 0.01
3 months	0.25 ± 0.01	0.25 ± 0.02	0.23 ± 0.01**	0.29 ± 0.02
6 months	0.29 ± 0.02	0.23 ± 0.02	0.24 ± 0.02*	0.30 ± 0.02
Glutathione peroxidase (U/mg Hb)				
Baseline (0 month)	0.74 ± 0.07	0.89 ± 0.06	0.90 ± 0.06	0.86 ± 0.06
3 months	0.87 ± 0.10	1.02 ± 0.15	0.88 ± 0.06	1.07 ± 0.08
6 months	0.88 ± 0.06	1.05 ± 0.13	1.19 ± 0.06*	1.12 ± 0.06*

Values are expressed as mean ± SEM.

Symbols indicate significant difference from baseline (0 month).

*: p < 0.05, **: p < 0.01

**Figure 1:**
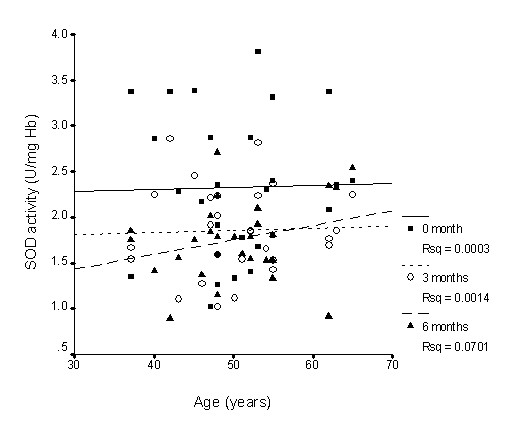
**Correlation between superoxide dismutase activity and age with TRF treatment for 6 months**. TRF supplementation strengthened the association after 6 months. 0 month: r = 0.04, p = 0.81; 3 months: r = 0.06, p = 0.73; 6 months: r = 0.26, p = 0.21.

**Figure 2:**
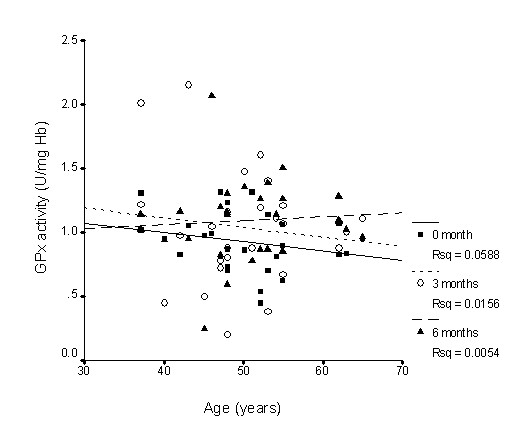
**Correlation between glutathione peroxidase activity and age with TRF treatment for 6 months**. TRF supplementation reversed the relationship after 6 months. 0 month: r = -0.17, p = 0.40; 3 months: r = -0.16, p = 0.41; 6 months: r = 0.05, p = 0.82.

**Figure 3:**
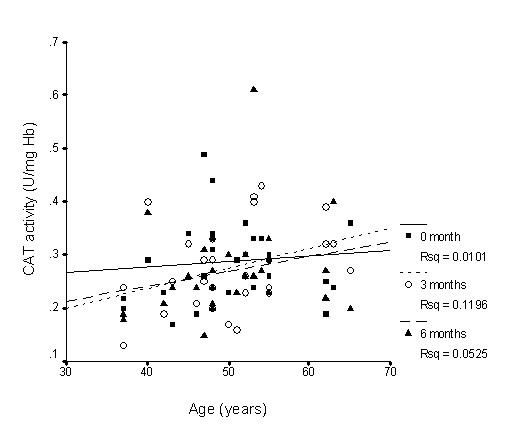
**Correlation between catalase activity and age with TRF treatment for 6 months**. TRF supplementation strengthened the association after 3 and 6 months. 0 month: r = 0.16, p = 0.41; 3 months: r = 0.36, p = 0.06; 6 months: r = 0.23, p = 0.26.

There was nearly no association between SOD activity and age (Figure 1), whereas GPx activity slightly declined with age (Figure 2), while CAT activity slightly increased with age (Figure 3); however, none of these correlations was significant. Supplementation with TRF strengthened the relationship between SOD activity and age as well as for CAT activity. GPx activity was reversed in the older individuals in association with age.

### Oxidative markers

Changes in protein carbonyl levels were statistically significant with duration of treatment (F = 6.193, p = 0.008, effect size = 0.212, power = 0.810). Protein carbonyl content was significantly decreased after 6 months of TRF treatment (p = 0.002) as compared to baseline (0 month) (Table 7). Further grouping by age (Table 8) revealed a marked reduction (p < 0.001) in the > 50 year-old group after 6 months of treatment. Nonetheless, no significant effect for duration was obtained in AGE and MDA levels despite both levels were reduced in the older TRF-treated group after 3 months and remained low thereafter; however, this tendency did not reach significance.

**Table 7:** Oxidative markers in placebo and TRF groups

	Duration					
Oxidative Markers	0 month		3 months		6 months	
	
	Placebo	TRF	Placebo	TRF	Placebo	TRF
Protein carbonyl (nmol/mg)	0.52 ± 0.05	0.63 ± 0.04	0.51 ± 0.05	0.53 ± 0.04	0.56 ± 0.05	0.45 ± 0.04**
Advanced glycosylation end-product (units/ml)	2.37 ± 0.11	2.38 ± 0.19	2.00 ± 0.15	2.40 ± 0.18	1.93 ± 0.17	1.92 ± 0.14
Malondialdehyde (nmol/ml)	4.21 ± 0.19	4.10 ± 0.30	4.00 ± 0.24	3.90 ± 0.25	3.91 ± 0.23	4.14 ± 0.22

Values are expressed as mean ± SEM.

Symbols indicate significant difference from baseline (0 month).

**: p < 0.01

**Table 8:** Oxidative markers according to age group

	Age group			
Oxidative markers	35-49 years		> 50 years	
	
	Placebo	TRF	Placebo	TRF
Protein carbonyl (nmol/mg)				
Baseline (0 month)	0.54 ± 0.08	0.58 ± 0.08	0.50 ± 0.06	0.63 ± 0.03
3 months	0.56 ± 0.13	0.54 ± 0.05	0.48 ± 0.05	0.52 ± 0.06
6 months	0.60 ± 0.08	0.51 ± 0.06	0.47 ± 0.04	0.40 ± 0.03**
Advanced glycosylation end-product (units/ml)				
Baseline (0 month)	2.39 ± 0.15	2.10 ± 0.22	2.34 ± 0.22	2.73 ± 0.26
3 months	2.01 ± 0.18	2.38 ± 0.21	2.30 ± 0.26	2.24 ± 0.35
6 months	2.12 ± 0.22	2.02 ± 0.17	1.98 ± 0.25	1.71 ± 0.24*
Malondialdehyde (nmol/ml)				
Baseline (0 month)	4.25 ± 0.30	3.45 ± 0.37	4.18 ± 0.23	4.71 ± 0.41
3 months	4.82 ± 0.34	3.95 ± 0.39	3.22 ± 0.22	3.86 ± 0.34
6 months	3.95 ± 0.31	4.48 ± 0.27	3.87 ± 0.36	3.87 ± 0.32

Values are expressed as mean ± SEM.

Symbols indicate significant difference from baseline (0 month).

*: p < 0.05, **: p < 0.001

**Figure 4:**
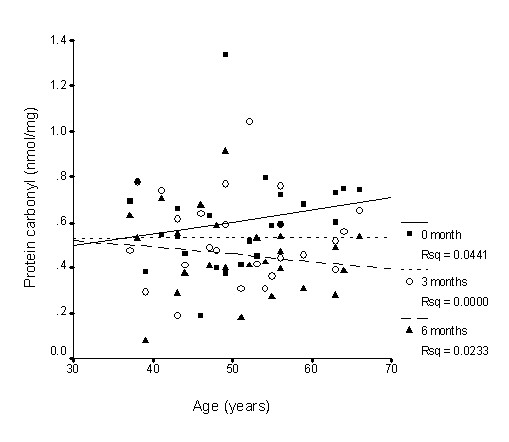
**Correlation between plasma protein carbonyl levels and age with TRF treatment for 6 months**. TRF supplementation weakened the association after 3 and 6 months. 0 month: r = 0.21, p = 0.31; 3 months: r = 0.004, p = 0.98; 6 months: r = -0.15, p = 0.47.

**Figure 5:**
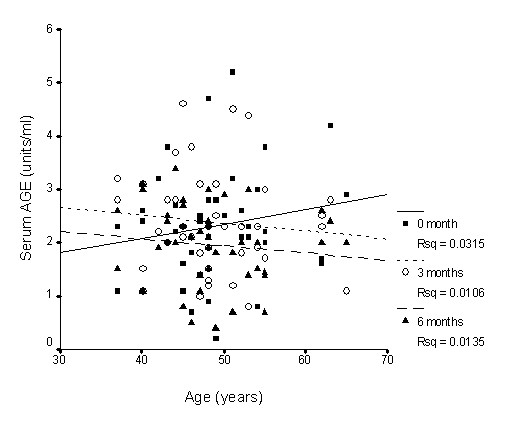
**Correlation between serum advanced glycosylation end-products and age with TRF treatment for 6 months**. TRF supplementation reversed the association after 3 and 6 months. 0 month: r = 0.18, p = 0.21; 3 months: r = -0.13, p = 0.37; 6 months: r = -0.12, p = 0.47.

**Figure 6:**
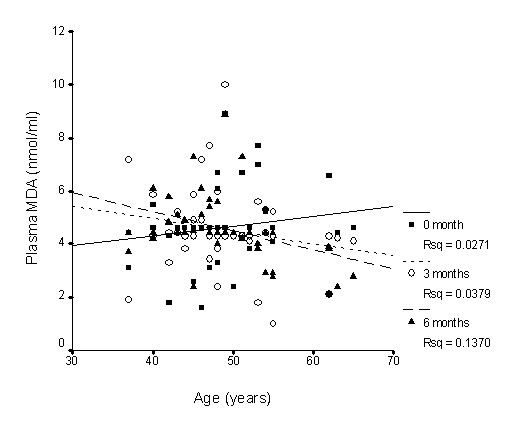
**Correlation between plasma malondialdehyde levels and age with TRF treatment for 6 months**. TRF supplementation reversed the association after 3 and 6 months. 0 month: r = 0.17, p = 0.25; 3 months: r = -0.19, p = 0.22; 6 months: r = -0.37, p = 0.02.

The relationships between age and oxidative stress marker levels are shown in Figures 4, 5 and 6. All measured biomarkers (protein carbonyl, AGE and MDA) were weakly correlated with age. TRF treatment reversed these relationships, particularly for MDA, which showed a correlation coefficient of close to 0.4 (p < 0.05).

## Discussion

Human ageing is affected by both genetic factors and lifestyle-related factors such as diet. Dietary intervention is feasible, as nutrients can affect the rate of ageing by altering the type and quantity of proteins synthesized [37] by modulating gene expression [38], thereby altering the oxidative status of individuals [39].

Our results indicate that daily supplementation for up to 6 months with TRF raised plasma HDL cholesterol levels as early as 3 months, thereby increasing the HDL-cholesterol/total cholesterol ratio. This ratio reflects the proportion of anti-atherogenic to atherogenic lipids and has been suggested as a better predictor of cardiovascular disease risk than the individual lipoprotein values [40]. TRF might thus help to reduce the risk of coronary heart disease (CHD) in healthy older adults. In fact, HDL cholesterol increases of the magnitude observed in this study have been associated with a 22.5% reduced risk of cardiovascular events [41]. Raising plasma HDL cholesterol and thus the HDL-cholesterol/total cholesterol is recommended by the American Diabetes Association (ADA) guidelines together with lowering plasma triglycerides for high-risk individuals particularly older adults as major mortality cases of CHD were 65 years old or older [42].

Conflicting data have been reported regarding effects of vitamin E supplementation which were mainly α-tocopherol on HDL cholesterol. Increased HDL cholesterol after α-tocopherol supplementation has been reported by some investigators [43-45] and disputed by others [46,47]. It should be noted that the supplement used in this study was high in tocotrienols, which has been reported to have different effect from α-tocopherol [48]. Tocotrienol but not tocopherol increases HDL cholesterol by inhibiting HMG-CoA reductase through signalling, thereby regulating cholesterol biosynthesis [49]. Tocotrienol may increase HDL in this study by modulating signal transduction and gene expression; specifically, and may normalize any aberrant gene expression incurred by aging [50]. Increases in HDL could be attained by increasing physical exercise [51,52] but similar effects by supplementation of vitamins have not been reported in human.

Compliance of the subjects was indicated by the observed increase in plasma lipid-corrected total tocotrienol and tocopherol concentration. Standardization of plasma vitamin E levels to total cholesterol was necessary to control for age-related changes in baseline cholesterol levels as vitamin E is transported by the lipoproteins. The finding that tocotrienol levels were increased is of particular interest, as tocotrienol is now reported to have functions distinct from α-tocopherol as reviewed by Sen *et al. *[53]. The marked increase in tocotrienol in the > 50 age group is interesting and suggests an increased bioavailability and possibly the need for tocotrienol with aging. The level of total tocotrienol was slightly lower in the older adults as opposed to the younger group. Supplementation of older subjects with TRF restored plasma vitamin E availability to near the levels of in the controls of the younger group. We speculate that a steady state plasma vitamin E concentration was achieved after 6 months of supplementation, as plasma concentrations were similar to those in the younger group were seen at that time point. Some studies have reported the achievement of steady-state plasma vitamin E levels after 10 to 15 days of supplementation with either natural or synthetic forms of α-tocopherol at much higher dosages [54-56]. Considering the well-documented preferential absorption and transportation of different vitamin E isomers in the body and by taking into account the tocotrienol-rich composition in the TRF, such a slow but steady increment is reasonable.

The tocotrienols are found in a wide variety of foods and it has been suggested recently that all 8 isomers of vitamin E may be necessary for optimum health [24]. This requirement maybe more crucial for the older individuals where digestion and absorption may not be as efficient resulting in the potential benefits of supplementation. Differences noted in the plasma levels of tocotrienols detected in various human studies are mainly due to the different daily fat diet. Asians consume higher levels of palm oil rich in tocotrienols. Therefore, a comparatively higher absorption of tocotrienol and thus better level found in circulation.

The elevated plasma vitamin C level detected in the present study was most likely absorbed from the diet, as the supplement is not a source of vitamin C. Indeed, we found a positive correlation between vitamin C intake and plasma vitamin C levels (r = 0.308, p = 0.048). This is in accordance with findings by Padayatty *et al. *[57] who had reported that small changes in oral intake of vitamin C resulted in large changes in plasma vitamin C concentration. The increase in plasma vitamin C might also result from a complementary effect by vitamin E in an interlinking antioxidant network. The involvement of vitamin C in regenerating vitamin E directly from its tocotrienoxyl or tocopheroxyl radical back to tocotrienol and tocopherol respectively has been well documented [58]. Increased levels of vitamin E might reflect increased reactions with reactive free radicals, additional formation of tocotrienoxyl or tocopheroxyl radical, and a further increased need for vitamin C.

The variation observed in enzyme activity might have been due to the different roles of the analysed antioxidant enzymes. The functional roles of these enzymes are well established, with SOD acting upstream by dismutating reactive superoxide anion radicals into more stable hydrogen peroxide (H_2_O_2_) whereas catalase and GPx function downstream by converting H_2_O_2 _into water and oxygen in apparently parallel pathways. Changes in antioxidant enzymes activity observed were clearly in favour of the > 50 years old group. Reduction in SOD activity with TRF supplementation after 3 and 6 months in the older group possibly due to lesser formation of radicals as a result of radical scavenging effect by tocotrienol and tocopherol. On the other hand, increase in GPx activity in both placebo and TRF group after 6 months possibly due to higher need for H_2_O_2 _removal. Increased level of tocotrienol and tocopherol attained by TRF supplementation might reflect more radical scavenging activity, followed by increased H_2_O_2 _formation and therefore increased requirement for its removal. As for the placebo group, increased intake of dietary vitamin C might results in a similar increase in radical scavenging activity and the subsequent reactions involving formation and detoxification of H_2_O_2_. Given a decline in the catalase activity in the placebo group, the action of removing H_2_O_2 _was predominantly done by GPx. In the current study, the enzymes activity measured was evidently influenced by TRF supplementation as shown by the shifted correlation patterns with age.

Long term supplementation of TRF for 6 months caused the increase in plasma vitamin E availability (both tocopherol and tocotrienol) observed in the current study, accompanied by changes in the oxidative stress biomarkers measured. Protein carbonyls have been described as oxidized amino acids resulting from direct oxidation of protein by reactive oxygen species [59]. Proteins are also modified indirectly by glycation or glycoxidation of amino groups with the eventual formation of the advanced glycosylation end products (AGEs) [60]. Consistent with the fall in plasma concentrations of carbonylated protein with TRF supplementation, a sharp decrease in serum AGE was observed. When the effect of age was factored out of the statistical model, it was found that the interaction between supplementation and duration was significant for the older individuals, indicating a favourable gain in the older group. Figures 4, 5 and 6 give individual presentations of the changes in these oxidative markers with age. Ascending trends of protein damage and lipid peroxidation accumulation during ageing as shown by the correlative data were reduced, even reversed by TRF supplementation. These findings confirm that nutritional intervention can exert cumulative effects on oxidative stress in healthy individuals in the long term [27]. The unique combination of vitamin E isomers used in the study might have acted synergistically to provide the beneficial effect.

The reduced levels of oxidative markers were mainly observed in the older group, for whom the lower cut-off point was 50 years of age. This is of interest as previous studies typically evaluated older subjects, mostly 60 years old and over. It is also noteworthy that in the present study the treatment was administered to healthy individuals for a lengthy period of 6 months and studies involving supplementation of this duration are fairly limited. This may then results in the observed changes in oxidative status as measured by protein carbonyl and AGE. Although some of the younger subjects in the study also showed increased levels of antioxidant vitamins, the magnitude of changes was less evident as compared to the older individuals. The absence or lack of response by the younger age group might reflect a well-maintained antioxidant level, more effective maintenance of oxidative balance, and better defence against spontaneous oxidative injury. It is thus possible that TRF supplementation did not provide any further improvement. Although baseline antioxidant levels in the > 50 year-old group were similar to those in the < 50 year-old group, baseline oxidative marker levels were higher in the older group, suggesting a higher level of oxidative damage. However, the amount of damaged lipids and proteins in this group was reduced by supplementation, probably due to the increased requirement for antioxidants in older individuals. It is possible that an antioxidant threshold for optimum performance exists and that this threshold (and therefore the requirement for antioxidants) could increase with ageing, thus allowing supplementation to generate an effect in the present study. A long-term prospective study will be required to test this hypothesis, particularly at the molecular level.

Compelling evidence suggests a new level of action for vitamin E under the non-antioxidative control in protection against disease [50]. Therefore, TRF might not only act directly or solely as an antioxidant, but it may actually also act through signalling pathways and specific signal-regulated protein reaction as suggested by Sen *et al. *[14,61]. Tocotrienol was shown to provide complete neuroprotection via antioxidant-independent mechanism with the protective property reported not only limited in response to non-oxidative challenges but also to oxidative insults [14,15]. Further studies of the effects of tocotrienols in a cell model are currently underway in our laboratory.

## Conclusion

Our data revealed an age-related increase in oxidative damage. We established a role of nutritional supplementation in oxidative damage and antioxidant levels in older individuals. To our best knowledge, tocotrienol-rich vitamin E supplementation has not yet been studied in relation to oxidative stress in healthy older individuals. Consistent with increased concentrations of plasma antioxidants (vitamins E and C), we observed significant decreases in protein carbonyl and AGE levels, as well as improvement of plasma cholesterol levels. The protective effects of TRF supplementation observed in this study might represent a restoration of redox balance, particularly in the > 50-year old group. Increased tocotrienol level might be an important mechanism by which TRF supplementation confers its protective benefits via protection against oxidative stress, involvement in oxidized protein repair, besides contributing to the regulation of redox homeostasis through signalling.

## Competing interests

The authors declare that they have no competing interests.

## Authors' contributions

SFC was involved in the acquisition, analysis and interpretation of data in addition to drafting the manuscript; JI, NAAH, AAL, ZZ and AAK made significant contributions to the acquisition of the data, SM was involved in interpretation of data and critical analysis of intellectual content of the manuscript, MM and YAMY contributed to the design, acquisition and interpretation of the data; and WZWN was instrumental in the study's inception, design and approval while providing critical analysis of data interpretation and manuscript review. The final manuscript have been read and approved by all authors.
